# Antibacterial Activity and Cellular Effects of Huai Chrysanthemum Polyphenol Extract Against Foodborne Pathogens

**DOI:** 10.3390/foods15183210

**Published:** 2026-09-11

**Authors:** Fuhua Zhang, Nan Wang, Shige Wen, Jiansheng Zhao, Mingwu Qiao, Lianjun Song, Xianqing Huang, Yan Ma

**Affiliations:** 1 Luohe Medical College, Luohe 462002, China; zhangfuhua@lhmc.edu.cn; 2 College of Food Science and Technology, Henan Agricultural University/Henan Agricultural University Key R&D Center of Meat Science and Industrial Technology, Zhengzhou 450002, China; 3 Henan Shuanghui Investment Development Co., Ltd./Henan Technology Innovation Center of Meat Processing and Research, Luohe 462000, China

**Keywords:** huai chrysanthemum polyphenol extract, phenolic acids, flavonoids, antibacterial mechanism, membrane damage, oxidative stress

## Abstract

Huai chrysanthemum (*Chrysanthemum morifolium* Ramat. cv. Huaiju), collected from Jiaozuo, Henan Province, China, is a medicinal and edible plant rich in phenolic compounds. Polyphenols are natural bioactive compounds with potential antibacterial activity. In this study, HPLC-MS/MS was used to identify and quantify the phenolic compounds in huai chrysanthemum polyphenol extract, and its antibacterial activity against representative Gram-negative and Gram-positive foodborne bacteria was evaluated. A total of 22 polyphenolic compounds were identified, including 10 phenolic acids, 10 flavonoids, and two other phenolic compounds. Caffeic acid was the predominant phenolic acid, while luteoloside was the predominant flavonoid. Flavonoids accounted for 94.03% of the total identified polyphenols. The extract inhibited the growth of both *Escherichia coli* and *Staphylococcus aureus*, with an MIC_80_ of 12.5 mg/mL for both strains. Further analysis showed that the extract reduced intracellular protein content and ATP levels, increased extracellular alkaline phosphatase activity, altered cell surface charge, affected superoxide dismutase activity, and caused marked morphological changes in bacterial cells. These results suggested that the antibacterial effect of huai chrysanthemum polyphenol extract was related to cell envelope damage, energy metabolism disturbance, and redox imbalance. This study provided a basis for further application of huai chrysanthemum polyphenol extract as a natural antibacterial ingredient in food preservation.

## 1. Introduction

Huai chrysanthemum, the dried capitulum of *Chrysanthemum morifolium* Ramat. cv. Huaiju, belonges to the Asteraceae family and represents a traditional medicinal and edible plant in China. It is mainly produced in Henan Province and is recognized as one of the four major Huai medicinal herbs. In traditional Chinese medicine, chrysanthemum flowers were used to disperse wind, clear heat, calm the liver, improve vision, and remove toxic heat [1,2]. The Pharmacopoeia of the People’s Republic of China recorded medicinal chrysanthemum flowers and listed several representative varieties, including bo chrysanthemum, chu chrysanthemum, gong chrysanthemum, hang chrysanthemum, and huai chrysanthemum [1]. Owing to its long history of consumption and pharmacological value, chrysanthemum was widely used in herbal tea, botanical beverages, functional foods, and health-related products [3]. Previous studies showed that chrysanthemum flowers contained abundant phenolic acids, flavonoids, carotenoids, and other bioactive compounds, which contributed to their antioxidant, anti-inflammatory, antimicrobial, hepatoprotective, and antihypertensive properties [4,5,6]. The biological activities of chrysanthemum flowers were closely associated with their phytochemical composition. Phenolic acids, flavonoids, carotenoids, volatile oils, polysaccharides, and other secondary metabolites were identified in different chrysanthemum species and cultivars. Among these constituents, caffeoylquinic acids and flavonoids were recognized as the principal bioactive components of *C. morifolium*. Phytochemical investigations identified chlorogenic acid, caffeic acid, dicaffeoylquinic acids, luteolin, apigenin, kaempferol, and their glycosides as major phenolic compounds in chrysanthemum flowers [7,8]. Yang et al. [9] further isolated new caffeoylquinic acid derivatives and a flavanone glycoside from *C. morifolium*, supporting the chemical diversity of this medicinal and edible flower. Related studies on edible flowers of the Asteraceae family also showed the value of flower-derived polyphenols as functional ingredients. For example, Siddiqa et al. [10] reported that Tagetes flowers contained abundant secondary metabolites and that extraction method and blooming stage affected their phenolic composition and antioxidant capacity. Another LC-ESI-QTOF-MS/MS-based study on Tagetes flowers identified 33 polyphenolic compounds and showed that freeze-dried samples extracted with 70% ethanol under ultrasound treatment had high total phenolic content and antioxidant activity [11].

Although chrysanthemum species and related Asteraceae flowers had been studied for their phytochemical profiles, antioxidant activities, and several pharmacological functions, the antibacterial mechanism of huai chrysanthemum polyphenol extract remained insufficiently clarified. Previous studies mainly focused on chemical characterization, antioxidant capacity, anti-inflammatory activity, hypoglycemic activity, hepatoprotective effects, or cultivar-level comparisons of total biological activities [8,12,13]. Some studies reported antimicrobial activities of chrysanthemum extracts or essential oils, but they often emphasized inhibition zones or MIC values rather than the physiological basis of bacterial damage [4,5]. For huai chrysanthemum specifically, limited information was available on whether its polyphenol extract affected bacterial membrane integrity, surface charge, intracellular protein leakage, ATP homeostasis, alkaline phosphatase release, oxidative stress response, and cell morphology. The limited mechanistic investigation of its antibacterial activity restricted the further evaluation and application of huai chrysanthemum polyphenol extract as a potential natural antimicrobial ingredient.

Plant polyphenols were widely recognized as secondary metabolites with antibacterial potential [14]. Their antibacterial effects were usually associated with interactions with bacterial cell envelopes, changes in membrane permeability, leakage of intracellular components, disturbance of energy metabolism, and oxidative-stress-related responses. Studies on other plant polyphenols, including flaxseed polyphenols, black raspberry root phenolics, *Perilla frutescens* and *Eugenia pyriformis* leaf extracts, showed inhibitory effects against foodborne or pathogenic bacteria, including *E. coli*, *S. aureus*, *Listeria monocytogenes*, and *Salmonella* spp. [15,16,17]. These findings provided a basis for investigating the antibacterial activity of huai chrysanthemum polyphenol extract.

Current studies on chrysanthemum mainly focused on chemical characterization, antioxidant capacity, anti-inflammatory activity, hypoglycemic activity, hepatoprotective effects, or cultivar-level comparisons [8,12,13]. Some studies reported antimicrobial activities of chrysanthemum extracts or essential oils, but most of them focused on inhibition zones or MIC values. For huai chrysanthemum, information on how its polyphenol extract affected bacterial envelope integrity, surface charge, intracellular protein leakage, ATP level, alkaline phosphatase release, oxidative-stress-related indicators, and cellular morphology remained limited. These indicators were closely related to bacterial physiological damage and were therefore suitable for evaluating the antibacterial action of huai chrysanthemum polyphenol extract.

*E. coli* and *S. aureus* were selected in this study as representative Gram-negative (Gram^−^) and Gram-positive (Gram^+^) bacteria, respectively. *E. coli* possessed an outer membrane rich in lipopolysaccharides, whereas *S. aureus* lacked an outer membrane but contained a thick peptidoglycan layer. These structural differences provided a useful model for comparing the antibacterial responses of bacteria with distinct envelope properties. Both bacteria were also commonly associated with food safety concerns and were frequently used in the evaluation of natural antibacterial substances.

This study prepared huai chrysanthemum polyphenol extract and characterized its phenolic constituents using high-performance liquid chromatography–tandem mass spectrometry. The antibacterial activity of the extract against *E. coli* and *S. aureus* was evaluated through inhibition-zone assay, MIC_80_ determination, and growth-curve analysis. The effects of the extract on bacterial envelope-related indicators, intracellular ATP content, oxidative-stress-related responses, cellular morphology, and intracellular protein profiles were further investigated. This study aimed to clarify the antibacterial activity of huai chrysanthemum polyphenol extract and to provide a basis for its development as a natural antibacterial ingredient for food-related applications.

## 2. Materials and Methods

### 2.1. Chemicals and Materials

Huai chrysanthemum samples, the dried capitula of *Chrysanthemum morifolium* Ramat. cv. Huaiju, were collected from Jiaozuo, Henan Province, China, in October 2025. The plant material was botanically authenticated by Fuhua Zhang at Scientific Compass (www.shiyanjia.com). The dried samples were stored at −20 °C before extraction. *E. coli* ATCC 25922, a Gram-negative (Gram^−^) reference strain, and *S. aureus* ATCC 25923, a Gram-positive (Gram^+^) reference strain, were obtained from Henan Agricultural University, Zhengzhou, Henan, China. The SOD detection kit and MDA detection kit were purchased from Beijing Solarbio Technology Co., Ltd., Beijing, China; the BCA protein concentration assay kit, ATP detection kit, and alkaline phosphatase assay kit were sourced from Shanghai Biotian Biological Technology Co., Ltd., Shanghai, China. All reagents were of domestic analytical grade, and the liquid chromatography reagents were of chromatographic grade.

### 2.2. Preparation of Chrysanthemum Extract

The extract was prepared according to Wang et al. [17] with slight modifications. Briefly, dried huai chrysanthemum flower heads were ground into powder, and 10 g of the powder was mixed with 100 mL of 80% ethanol (solid-to-liquid ratio of 1:10, *w*/*v*). The mixture was stirred at 1000 rpm for 1 h at room temperature and centrifugation at 8000× *g* for 10 min. The residue was extracted three times under the same conditions. The supernatants were collected, combined, and concentrated under reduced pressure. The concentrate was frozen at −80 °C and then lyophilized under vacuum using a freeze dryer. The resulting powder was designated as huai chrysanthemum polyphenol extract.

### 2.3. Analysis of Polyphenol Extracts from Chrysanthemum

Liquid chromatography–mass spectrometry (LC–MS) analysis of the chrysanthemum polyphenol extract was performed according to the method of Wang et al. [17] with slight modifications. Huai chrysanthemum powder (1.0 g) was mixed with 20 mL of 80% methanol and extracted by ultrasonication for 30 min. The mixture was then centrifuged at 10,000× *g* for 10 min, and the supernatant was collected. The residue was extracted three additional times under the same conditions. The combined supernatants were concentrated under reduced pressure at 40 °C to a final volume of approximately 3–5 mL, reconstituted to 10 mL with 50% methanol, and filtered through a 0.45 μm organic membrane prior to UPLC–MS analysis.

Chromatographic separation was performed on an ultra-performance liquid chromatography system (Vanquish UPLC, Thermo Fisher Scientific, Waltham, MA, USA) equipped with a Waters HSS T3 column (50 × 2.1 mm, 1.8 μm, Milford, MA, USA). The mobile phase consisted of ultrapure water containing 0.1% acetic acid (solvent A) and acetonitrile containing 0.1% acetic acid (solvent B). The flow rate was set at 0.30 mL/min, the column temperature was maintained at 40 °C, and the injection volume was 2 μL. The gradient elution program was as follows: 0.0 min, A/B = 90:10 (V/V); 2.0 min, A/B = 90:10; 6.0 min, A/B = 40:60; 8.0 min, A/B = 40:60; 8.1 min, A/B = 90:10; and 12.0 min, A/B = 90:10. Throughout the analysis, samples were maintained at 4 °C in the autosampler.

Mass spectrometric detection was carried out using a high-resolution mass spectrometry system (Q Exactive, Thermo Fisher Scientific, USA) equipped with an electrospray ionization (ESI) source. The operating parameters were set as follows: sheath gas, 40 arb; auxiliary gas, 10 arb; spray voltage, −2.8 kV; auxiliary gas heater temperature, 350 °C; and capillary temperature, 320 °C. Data were acquired in negative ion mode using selected ion monitoring (SIM), with a full scan *m*/*z* range of 100–900.

### 2.4. Determination of Bacterial Activity of Polyphenol Extracts from Chrysanthemum

#### 2.4.1. Microbial Cultivation

*E. coli* and *S. aureus* were inoculated into LB medium and cultured in a shaking incubator (200 rpm, 37 °C) until the logarithmic growth phase was reached (OD_600_nm = 0.6). The bacterial suspension was then retained for further use.

#### 2.4.2. Determination of Antibacterial Activity of Chrysanthemum Polyphenol Extract

The Oxford cup method was used to evaluate the antimicrobial activity of huai chrysanthemum polyphenol extract against pathogenic bacteria, following the method described by Wang et al. [17] with slight modifications. A 200 μL aliquot of the bacterial suspension, cultured to the logarithmic phase (bacterial density 10^6^–10^8^ CFU/mL), was spread on agar plates. Sterile Oxford cups [6 × 7.8 × 10 mm (inner diameter × outer diameter × height)] were placed on the inoculated agar plates to prepare wells, and 100 μL of huai chrysanthemum polyphenol extract at concentrations of 50, 25, 12.5, 6.25, and 3.125 mg/mL was added to each well. The plates were incubated at 37 °C for 18 h. Sterile water was used as a negative control, and 50 μg/mL kanamycin was used as a positive control. After adding the samples, the Petri dish was placed at room temperature for 30 min, and then put it in the 37 °C incubator. The presence and diameter of the inhibition zones were observed and recorded as an indicator of bacterial sensitivity. The diameters of each inhibition zone were measured using a vernier caliper, with three replicates for each treatment group.

#### 2.4.3. Determinations of Minimum Inhibitory Concentration

Following the method of Akarca [18] with modifications, bacterial suspensions were prepared from logarithmic-phase cultures and adjusted to an OD_600_ of 0.6. The extract stock solution was prepared at 100 mg/mL in LB medium and then serially diluted with LB medium to obtain working solutions at twice the desired final concentrations. For the treatment wells, 100 μL of bacterial suspension was mixed with 100 μL of extract solution in 96-well plates, resulting in final extract concentrations of 50.00, 25.00, 12.50, 6.25, and 3.125 mg/mL. Wells containing 100 μL of bacterial suspension and 100 μL of LB medium without extract served as the growth control. To correct for the background absorbance of the extract, sample blanks were prepared by mixing 100 μL of each extract solution with 100 μL of LB medium without bacterial inoculation. LB medium without bacteria or extract was used as the medium blank. Each treatment was performed with six replicate wells. The plates were incubated at 37 °C for 24 h, and the absorbance was measured at 600 nm using a microplate reader (BioTek Instruments, Inc., Winooski, VT, USA). The bacterial growth-inhibition rate was calculated as follows:

$$Inhibition\;rate(\%)=[1-\frac{{OD}_{sample}-{OD}_{sample\;blank}}{{OD}_{growth\;control}-{OD}_{medium\;blank}}]\times 100$$

where ${OD}_{sample}{,\;OD}_{sample\;blank}{,\;OD}_{growth\;control}{,\;OD}_{medium\;blank}$ corresponded to extract-treated bacterial cultures, extract-containing medium without bacteria, untreated bacterial cultures, and LB medium alone, respectively. MIC_80_ was defined as the lowest final extract concentration that inhibited at least 80% of bacterial growth.

#### 2.4.4. Effect of Chrysanthemum Polyphenol Extract on the Growth Curve of Pathogenic Bacteria

Following the method of Kanatt, Arjun, & Sharma [19], 50 mL of *E. coli* and *S. aureus* cultures, grown to the logarithmic phase, were treated with different concentrations of chrysanthemum polyphenol extract, achieving final concentrations of 0 (control), MIC_80_, and 2 MIC_80_. A positive control of 50 μg/mL kanamycin was used. The cultures were incubated at 37 °C with shaking at 200 rpm. Samples were taken every h starting from 0 h monitored for 24 h, and OD_600_ nm was measured. Growth curves were plotted based on the relationship between incubation time and OD_600_ nm.

#### 2.4.5. Determination of Bacterial Cell Membrane Integrity

Following the method of Wang et al. [20], *E. coli* and *S. aureus* cultures, grown to the logarithmic phase, were centrifuged at 5000× *g* for 5 min at 4 °C. The bacterial pellets were washed twice with sterile water and then resuspended to a concentration of 10^7^ CFU/mL. The bacterial suspensions were mixed with chrysanthemum polyphenol extract at a 1:1 ratio, achieving final concentrations of MIC_80_ and 2 MIC_80_. The mixtures were incubated at 37 °C for 6 h, and samples were taken every h. After incubation, the mixtures were centrifuged to remove the bacterial cells, and the supernatants were filtered through a 0.22 μm cellulose acetate membrane. Protein concentrations in the filtrates were then measured according to the instructions of the BCA protein assay kit.

#### 2.4.6. Determination of Bacterial Cell Surface Zeta Potential

Following the method of Wang et al. [21] with modifications, Bacterial suspensions were adjusted to approximately 10^6^–10^7^ CFU/mL, *E. coli* and *S. aureus* suspensions were mixed with chrysanthemum polyphenol extract at a 1:1 ratio to achieve final concentrations of MIC_80_ and 2 MIC_80_. After incubating at 37 °C for 12 h, After treatment at 37 °C for 12 h, bacterial cells were harvested by centrifugation at 5000× *g* for 10 min at 4 °C and then washed twice with sterile phosphate-buffered saline. The treated bacterial strains were then resuspended in sterile water and analyzed using a nanoparticle size and Zeta potential(mV) (Malvern Panalytical Ltd., Malvern, Worcestershire, UK) analyzer.

#### 2.4.7. Determination of Intracellular ATP Content in Bacterial Cells

Following the method of Wang et al. [22] with modifications, *E. coli* and *S. aureus* cultures, grown to the logarithmic phase, were centrifuged at 5000× *g* for 5 min at 4 °C. The bacterial pellets were washed twice with sterile water and resuspended to the same OD_600_ value. The corresponding bacterial cell number was estimated according to the OD_600_-based conversion method provided in the kit instructions. The bacterial suspensions were mixed with chrysanthemum polyphenol extract at a 1:1 ratio, achieving final concentrations of MIC_80_ and 2 MIC_80_. The mixtures were then incubated at 37 °C for 30 min. Samples were processed according to the instructions of the ATP detection kit, and the intracellular ATP content in the bacterial cells was measured. Briefly, bacterial pellets were lysed with the extraction buffer provided in the kit, and the supernatant was collected after centrifugation. The absorbance/luminescence was measured using a microplate reader. ATP levels were expressed as μmol/10^6^ cell.

#### 2.4.8. Determination of Extracellular Alkaline Phosphatase Activity in Bacterial Cells

*E. coli* and *S. aureus* cultures, grown to the logarithmic phase, were centrifuged at 5000× *g* for 5 min at 4 °C. The bacterial pellets were washed twice with sterile water and resuspended to a concentration of 10^7^ CFU/mL. The bacterial suspensions were mixed with chrysanthemum polyphenol extract at a 1:1 ratio, achieving final concentrations of MIC_80_ and 2 MIC_80_. The mixtures were then incubated at 37 °C for 12 h. Every 3 h, the alkaline phosphatase activity in the cell-free supernatant was measured according to the instructions of the assay kit.

#### 2.4.9. Determination of SOD and MDA Levels in Bacterial Cells

Following the method of Weng et al. [23] with modifications, SOD activity and MDA content in bacterial cells were determined using commercial assay kits according to the manufacturer’s instructions with slight modifications. *E. coli* and *S. aureus* were cultured to the logarithmic phase and adjusted to the same OD_600_ value. The corresponding bacterial cell number was estimated according to the OD_600_-based conversion method provided in the kit instructions. The bacterial suspensions were mixed with huai chrysanthemum polyphenol extract to achieve final concentrations of 1/2MIC_80_, MIC_80_, and 2MIC_80_. The group without polyphenol extract served as the untreated control. The mixtures were incubated at 37 °C with shaking at 200 rpm. At each sampling time, 8 mL of bacterial suspension was centrifuged at 6000× *g* for 10 min at 4 °C. The supernatant was discarded, and the bacterial pellets were washed three times with PBS. The pellets were resuspended in PBS and disrupted on ice to obtain bacterial homogenates. The homogenates were centrifuged at 4500× *g* for 10 min, and the supernatants were analyzed for SOD activity and MDA content using the corresponding assay kits. The results were normalized to the estimated bacterial cell number and expressed as U/10^4^ cells for SOD activity and nmol/10^6^ cells for MDA content.

#### 2.4.10. Observation of Bacterial Cell Morphology

Following the method of Zhang et al. [24] with modifications, *E. coli* and *S. aureus* were cultured to the logarithmic phase and centrifuged at 5000× *g* for 5 min at 4 °C. The bacterial pellets were washed twice with sterile water and resuspended to a concentration of 10^7^ CFU/mL. The bacterial suspensions were mixed with chrysanthemum polyphenol extract at a 1:1 ratio to achieve final concentrations of MIC_80_ and 2 MIC_80_, and incubated at 37 °C for 12 h. After incubation, the mixtures were centrifuged at 8000× *g* for 10 min at 4 °C, and the pellets were washed with PBS buffer.

For scanning electron microscopy (SEM), the bacterial pellets were fixed overnight at 4 °C in 2.5% glutaraldehyde solution. The samples were then dehydrated using an ethanol gradient (30%, 50%, 70%, 80%, 90%, 100%, and 100% ethanol), with each step lasting 15 min. After dehydration, the samples were treated with isoamyl acetate for solvent exchange and dried. The specimens were gold-coated for 30 s before being observed under SEM to examine the microscopic morphology.

For transmission electron microscopy (TEM), the bacterial preparation followed the same steps as for SEM up to the fixation stage. The bacterial suspension was fixed overnight at 4 °C with 2.5% glutaraldehyde, followed by fixation with 1% osmium tetroxide for 3 h. The samples were then washed three times with PBS and dehydrated through a series of acetone solutions (30%, 50%, 70%, 80%, 90%, 95%, and 100%). The samples were then embedded, sectioned, and double-stained with uranyl acetate and lead citrate before being observed under TEM.

#### 2.4.11. Sodium Dodecyl Sulfate–Polyacrylamide Gel Electrophoresis

Following the method of Li et al. [25], sodium dodecyl sulfate–polyacrylamide gel electrophoresis (SDS-PAGE) was performed. *E. coli* and *S. aureus* were cultured to the logarithmic phase and centrifuged at 5000× *g* for 5 min at 4 °C. The bacterial pellets were washed twice with sterile water and resuspended to a concentration of 10^7^ CFU/mL. The bacterial suspensions were mixed with chrysanthemum polyphenol extract at a 1:1 ratio to achieve a final concentration of MIC_80_. Equal volumes of sterile water and 50 μg/mL kanamycin were used as negative and positive controls, respectively. The mixtures were incubated at 37 °C for 12 h. Every 3 h, the bacterial suspensions were centrifuged, washed twice, and resuspended in sterile water (10^7^ CFU/mL). A 5 μL aliquot of the bacterial suspension was mixed with an equal volume of loading buffer and heated at 100 °C for 10 min. Electrophoresis was performed using a 12% separating gel and a 5% stacking gel. The gel was stained with 0.1% Coomassie Brilliant Blue R250, and after decolorization, the gel was observed and photographed using a gel imaging system (Bio-Rad Laboratories, Inc., Hercules, CA, USA).

### 2.5. Statistical Analysis

All data were first assessed for normality using the Shapiro–Wilk test before statistical analysis. Each experiment was performed three independent biological replicates. Statistical analysis was conducted using one-way analysis of variance (ANOVA) with SPSS 20.0 software (SPSS Inc., Chicago, IL, USA). Data were expressed as the mean ± standard deviation (SD). Significant differences among treatments were determined using Duncan’s multiple range test, with *p* < 0.05 considered statistically significant.

## 3. Results

### 3.1. Phenolic Composition of Huai Chrysanthemum Polyphenol Extract

**Figure 1:**
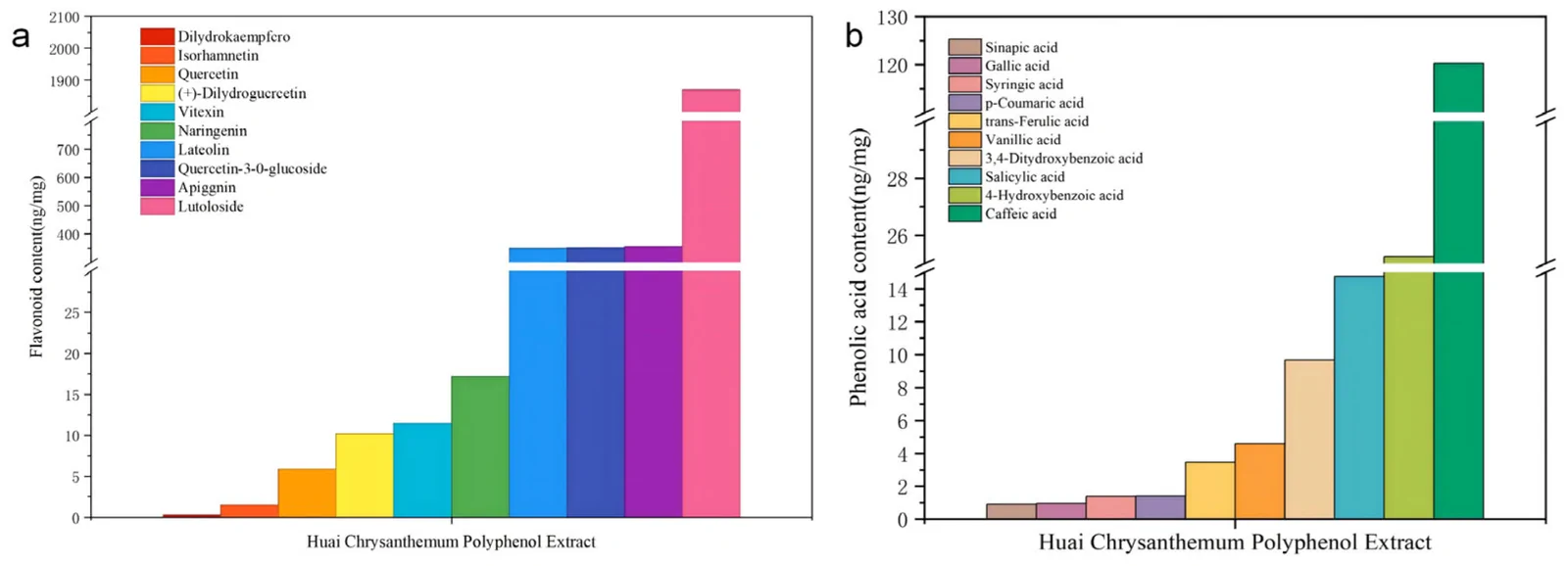
Composition of polyphenolic compounds in huai chrysanthemum polyphenol extract. (**a**) Composition and relative proportions of individual flavonoids. (**b**) Composition and relative proportions of individual phenolic acids. The content of each compound was shown as ng/mg extract.

The phenolic composition of huai chrysanthemum polyphenol extract was analyzed by LC–MS/MS. As shown in Figure 1, a total of 22 phenolic compounds were identified and quantified, including 10 phenolic acids, 10 flavonoids, and two other phenolic compounds. The identified phenolic acids included caffeic acid, syringic acid, p-coumaric acid, salicylic acid, trans-ferulic acid, sinapic acid, gallic acid, protocatechuic acid, vanillic acid, and 4-hydroxybenzoic acid. The identified flavonoids included luteolin, quercetin-3-O-glucoside, quercetin, luteoloside, vitexin, isorhamnetin, naringenin, dihydroquercetin, apigenin, and dihydrokaempferol. Resveratrol and syringaldehyde were detected as two other phenolic compounds.

Flavonoids were the predominant class of phenolic compounds in the extract, accounting for 94.03% of the total identified polyphenols. Among the flavonoids, luteoloside was the most abundant compound, with a content of 1869.95 ng/mg extract, accounting for 62.92% of the total flavonoids. Apigenin, quercetin-3-O-glucoside, and luteolin were also present at relatively high levels, accounting for 11.96%, 11.80%, and 11.76% of the total flavonoids, respectively. Among the phenolic acids, caffeic acid was the predominant compound, with a content of 120.24 ng/mg extract.

### 3.2. Antibacterial Activity and MIC_80_ of Huai Chrysanthemum Polyphenol Extract

**Figure 2:**
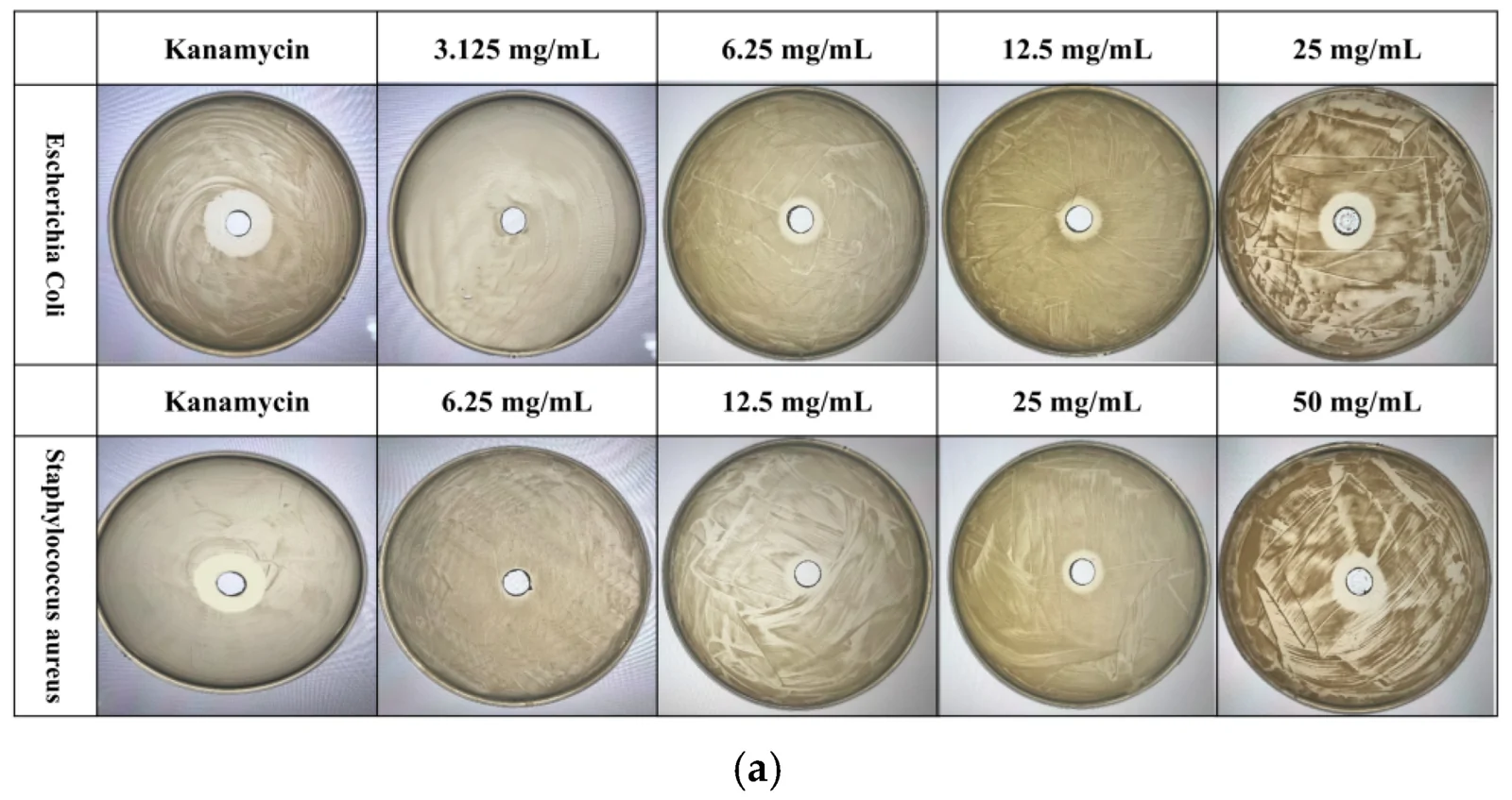

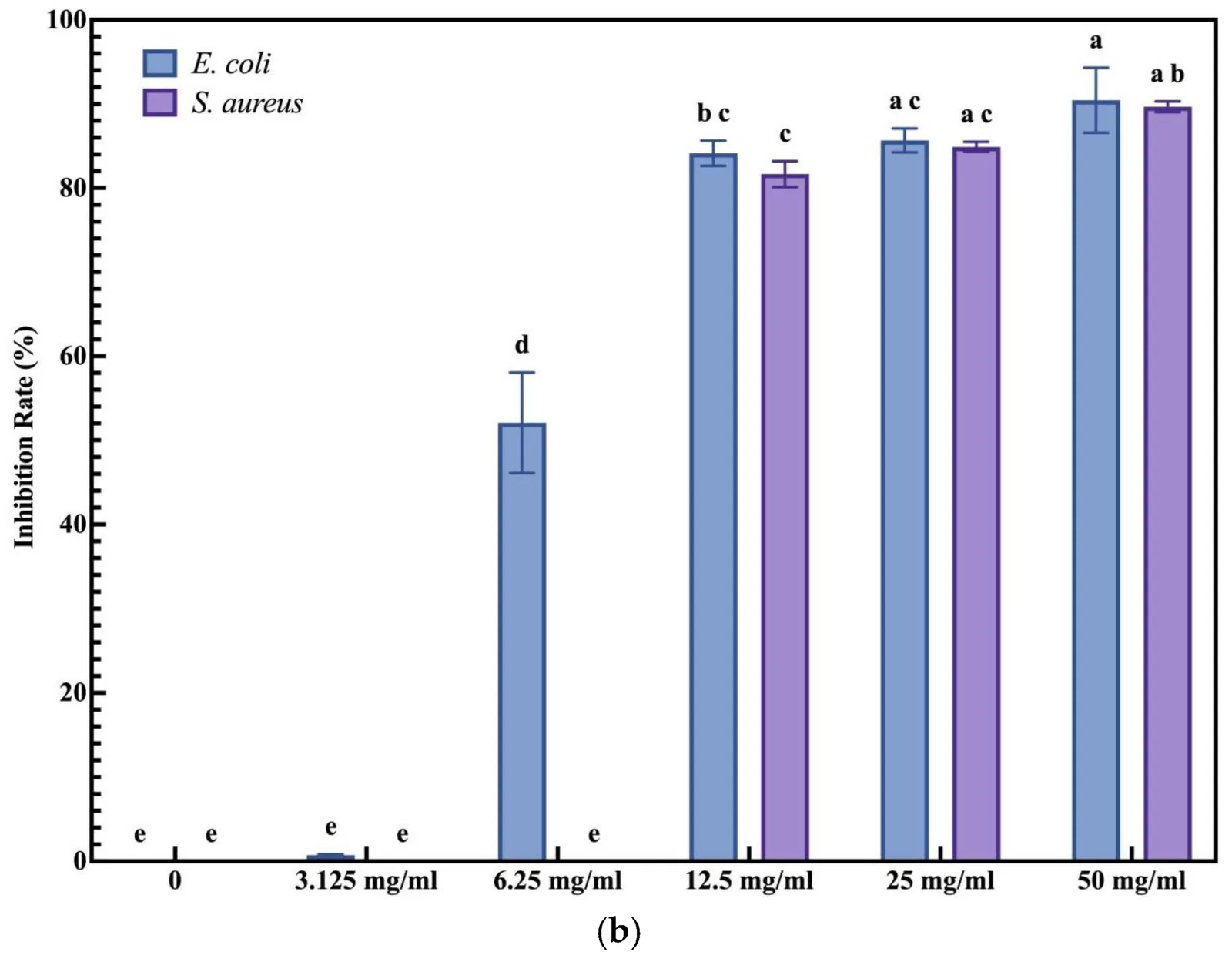
Antibacterial activity of huai chrysanthemum polyphenol extract against *E. coli* and *S. aureus*. (**a**) Inhibition-zone assay and (**b**) determination of the minimum inhibitory concentration (MIC_80_). MIC_80_ is defined as the lowest concentration that inhibited at least 80% of bacterial growth under the present experimental conditions. Data are expressed as the mean ± SD. Statistical analysis was performed by one-way analysis of variance (ANOVA), followed by Duncan’s multiple range test. Different lowercase letters indicate significant differences among groups (*p* < 0.05).

The antibacterial activity of huai chrysanthemum polyphenol extract was first evaluated using the Oxford cup assay. As shown in Figure 2a, clear inhibition zones were observed on plates inoculated with *E. coli* and *S. aureus* after treatment with the extract. The diameter of the inhibition zone increased with increasing extract concentration. At 25 mg/mL, the inhibition zone diameter reached 22.97 mm for *E. coli* and 16.48 mm for *S. aureus*.

The growth-inhibition assay further confirmed the antibacterial effect of the extract (Figure 2b). The inhibition rates of both strains increased as the extract concentration increased. For *E. coli*, the inhibition rate reached 51.52% at 6.25 mg/mL and increased to 84.14% at 12.5 mg/mL. For *S. aureus*, no obvious inhibition was observed at 3.125 and 6.25 mg/mL, whereas the inhibition rate reached 81.65% at 12.5 mg/mL. The inhibition rates in the 12.5, 25, and 50 mg/mL groups were significantly higher than those in the CK and low-concentration groups (*p* < 0.05). Based on the 80% growth-inhibition criterion, the MIC_80_ values of huai chrysanthemum polyphenol extract against both *E. coli* and *S. aureus* were determined to be 12.5 mg/mL.

### 3.3. Effects of Huai Chrysanthemum Polyphenol Extract on Bacterial Growth Curves

**Figure 3:**
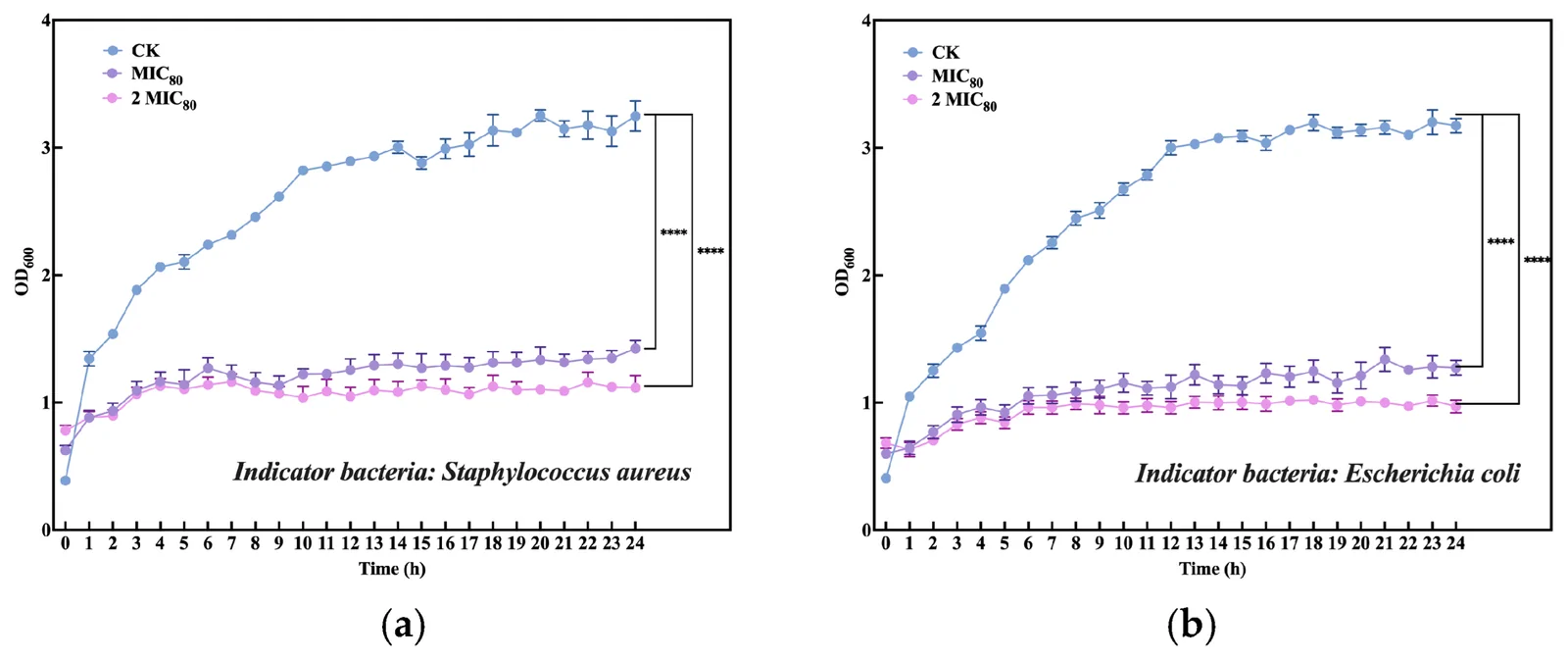
Effects of huai chrysanthemum polyphenol extract on the growth curves of *S. aureus* (**a**) and *E. coli* (**b**). **** indicates significant differences among the groups treated with different concentrations of chamomile polyphenols. The same statistical analysis as described in Figure 2 was applied at each time point.

The effects of huai chrysanthemum polyphenol extract on the growth of *E. coli* and *S. aureus* were further evaluated by monitoring OD_600_ values during incubation. As shown in Figure 3, the OD_600_ values of the control groups increased rapidly after the initial lag phase, indicating normal bacterial proliferation. In contrast, treatment with huai chrysanthemum polyphenol extract slowed the increase in OD_600_ values in both bacterial strains. Compared with the control group, the MIC_80_ treatment delayed the logarithmic growth phase and reduced bacterial growth during incubation. The inhibitory effect was more evident in the 2MIC_80_ group, in which OD_600_ values remained lower than those in the MIC_80_ group throughout most of the monitored period.

For both *E. coli* and *S. aureus*, the growth curves showed a concentration-dependent inhibitory pattern. The 2MIC_80_ treatment produced stronger growth suppression than the MIC_80_ treatment, suggesting that increasing extract concentration enhanced the antibacterial effect. These results were consistent with the inhibition-zone and MIC_80_ results, further confirming that huai chrysanthemum polyphenol extract inhibited the proliferation of both Gram-negative and Gram-positive bacteria under the present experimental conditions.

### 3.4. Effects of Huai Chrysanthemum Polyphenol Extract on Bacterial Cell Membranes

**Figure 4:**
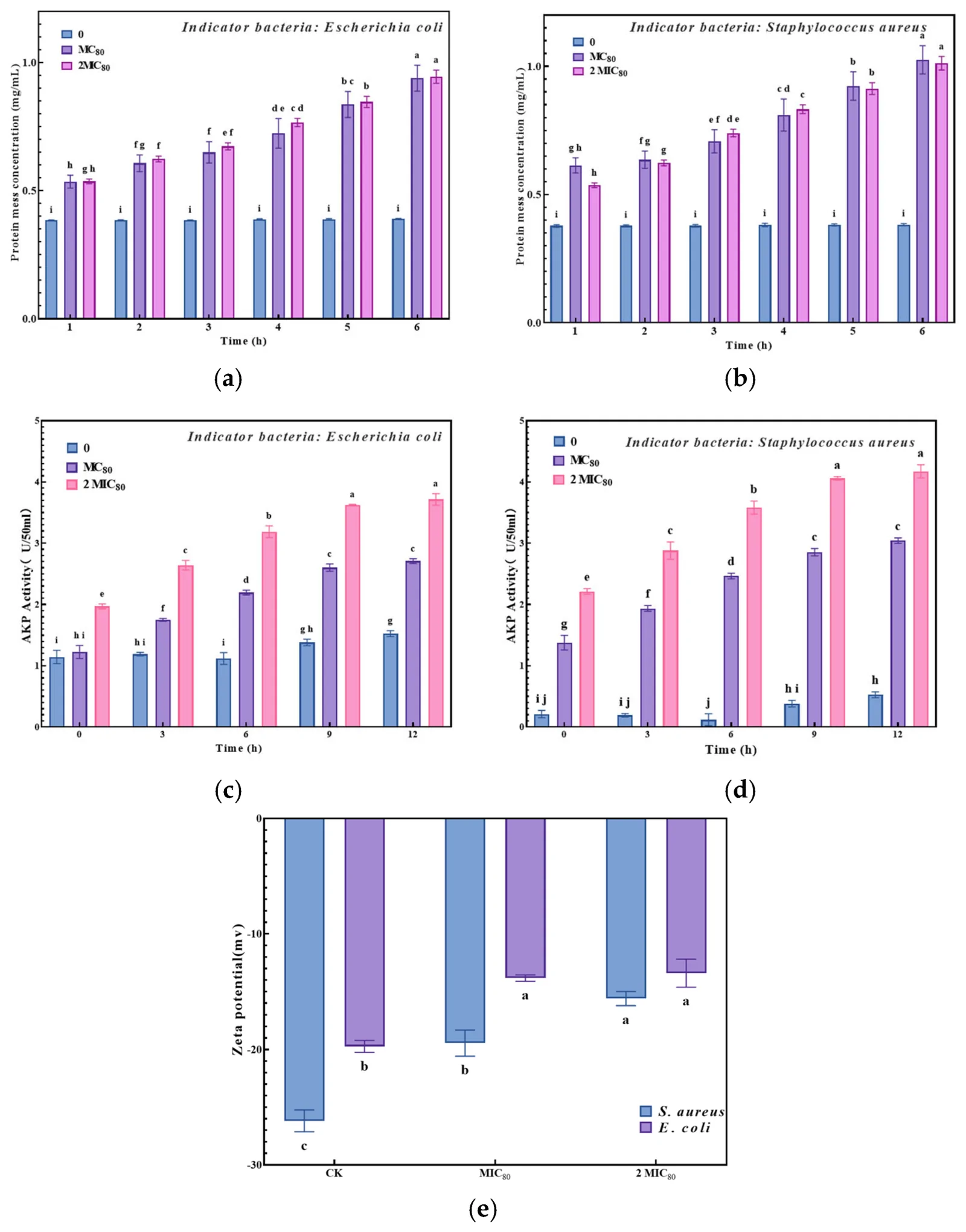
Effects of huai chrysanthemum polyphenol extract on bacterial cell membranes. Treatment with chrysanthemum polyphenol extract on protein leakage in pathogenic bacteria (**a**,**b**), extracellular alkaline phosphatase (**c**,**d**), zeta potential (**e**). The same statistical analysis as described in Figure 2 was applied. Different lowercase letters indicate significant differences among groups (*p* < 0.05).

Extracellular protein leakage, cell surface zeta potential, and extracellular alkaline phosphatase (AKP) activity were measured to evaluate the effects of huai chrysanthemum polyphenol extract on bacterial cell envelope-related properties. As shown in Figure 4a,b, the protein concentration in the culture supernatant increased after treatment with the extract. For *E. coli*, extracellular protein concentration increased with treatment time and extract concentration. Compared with the control group, both MIC_80_ and 2MIC_80_ treatments significantly increased extracellular protein leakage at all time points (*p* < 0.05). The protein concentration increased from approximately 0.53 mg/mL at 1 h to 0.94–0.96 mg/mL at 6 h in the treated groups. A similar trend was observed for *S. aureus*, and the extracellular protein concentration in the MIC_80_ and 2MIC_80_ groups was significantly higher than that in the control group throughout the treatment period (*p* < 0.05), reaching approximately 1.00 mg/mL at 6 h.

The bacterial cell wall played an important role in maintaining cell morphology and protecting cells against external stress. Alkaline phosphatase (AKP) activity in the culture supernatant was commonly used as an indicator of changes in cell envelope permeability. Under normal conditions, AKP was retained within bacterial cells or the periplasmic region. When the cell envelope was damaged, AKP could be released into the extracellular environment, leading to increased AKP activity in the culture supernatant [23,26]. Extracellular AKP activity was also increased after treatment with the extract (Figure 4c,d). In *E. coli*, AKP activity increased with treatment time and extract concentration. Compared with the control group, MIC_80_ treatment significantly increased extracellular AKP activity from 3 h onward, while 2MIC_80_ treatment resulted in a further increase at each time point (*p* < 0.05). At 12 h, AKP activity reached approximately 2.7 U/50 mL in the MIC_80_ group and 3.7 U/50 mL in the 2MIC_80_ group. A similar concentration- and time-dependent increase was observed in *S. aureus*. At 12 h, AKP activity reached approximately 3.0 and 4.2 U/50 mL in the MIC_80_ and 2MIC_80_ groups, respectively.

Changes in bacterial surface charge were evaluated by measuring zeta potential [27]. As shown in Figure 4e, both *S. aureus* and *E. coli* exhibited negative zeta potential values under the present experimental conditions. After treatment with huai chrysanthemum polyphenol extract, the zeta potential values shifted toward less negative values. For *S. aureus*, the zeta potential increased from approximately −26 mV in the CK group to approximately −19 mV and −16 mV in the MIC_80_ and 2MIC_80_ groups, respectively, with significant differences among groups (*p* < 0.05). For *E. coli*, the zeta potential changed from approximately −20 mV in the CK group to approximately −14 mV after MIC_80_ and 2MIC_80_ treatments. Both treatment groups differed significantly from the CK group, whereas no significant difference was observed between the MIC_80_ and 2MIC_80_ groups.

### 3.5. Effects of Huai Chrysanthemum Polyphenol Extract on Intracellular ATP Content

**Figure 5:**
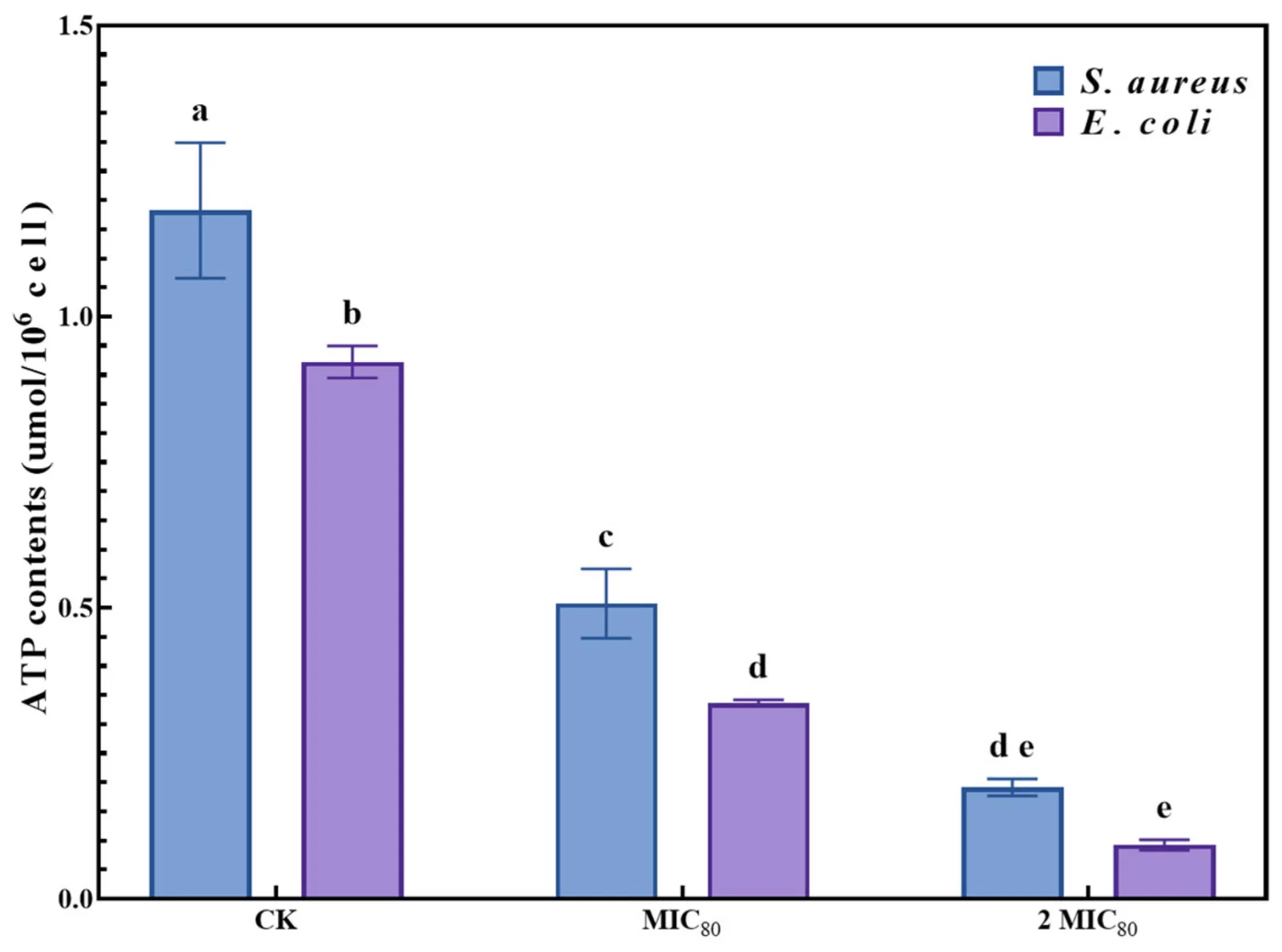
Effects of huai chrysanthemum polyphenol extract on intracellular ATP content in *S. aureus* and *E. coli*. The same statistical analysis as described in Figure 2 was applied. Different lowercase letters indicate significant differences among groups (*p* < 0.05).

Intracellular ATP content was measured to evaluate changes in bacterial energy-related metabolism after treatment with huai chrysanthemum polyphenol extract. As shown in Figure 5, the extract significantly reduced intracellular ATP levels in both *E. coli* and *S. aureus*. Compared with the CK group, ATP levels decreased markedly after MIC_80_ treatment and were further reduced after 2MIC_80_ treatment (*p* < 0.05). In *S. aureus*, ATP content decreased from approximately 1.18 μmol/10^6^ cells in the CK group to 0.50 and 0.19 μmol/10^6^ cells in the MIC_80_ and 2MIC_80_ groups, respectively. In *E. coli*, ATP content decreased from approximately 0.92 μmol/10^6^ cells to 0.33 and 0.09 μmol/10^6^ cells, respectively. These results indicated that huai chrysanthemum polyphenol extract reduced intracellular ATP levels in a concentration-dependent manner.

### 3.6. Effects of Huai Chrysanthemum Polyphenol Extract on Bacterial Cell Oxidative Stress

**Figure 6:**
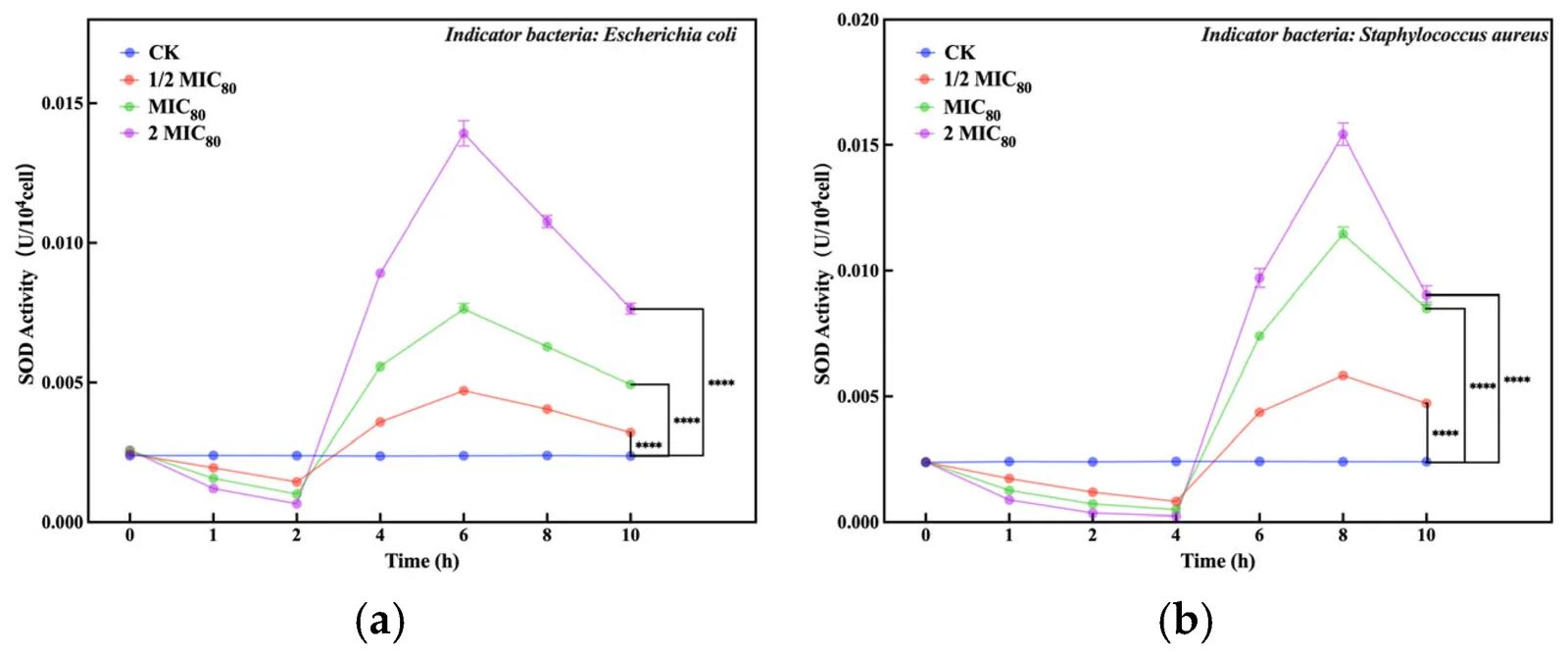

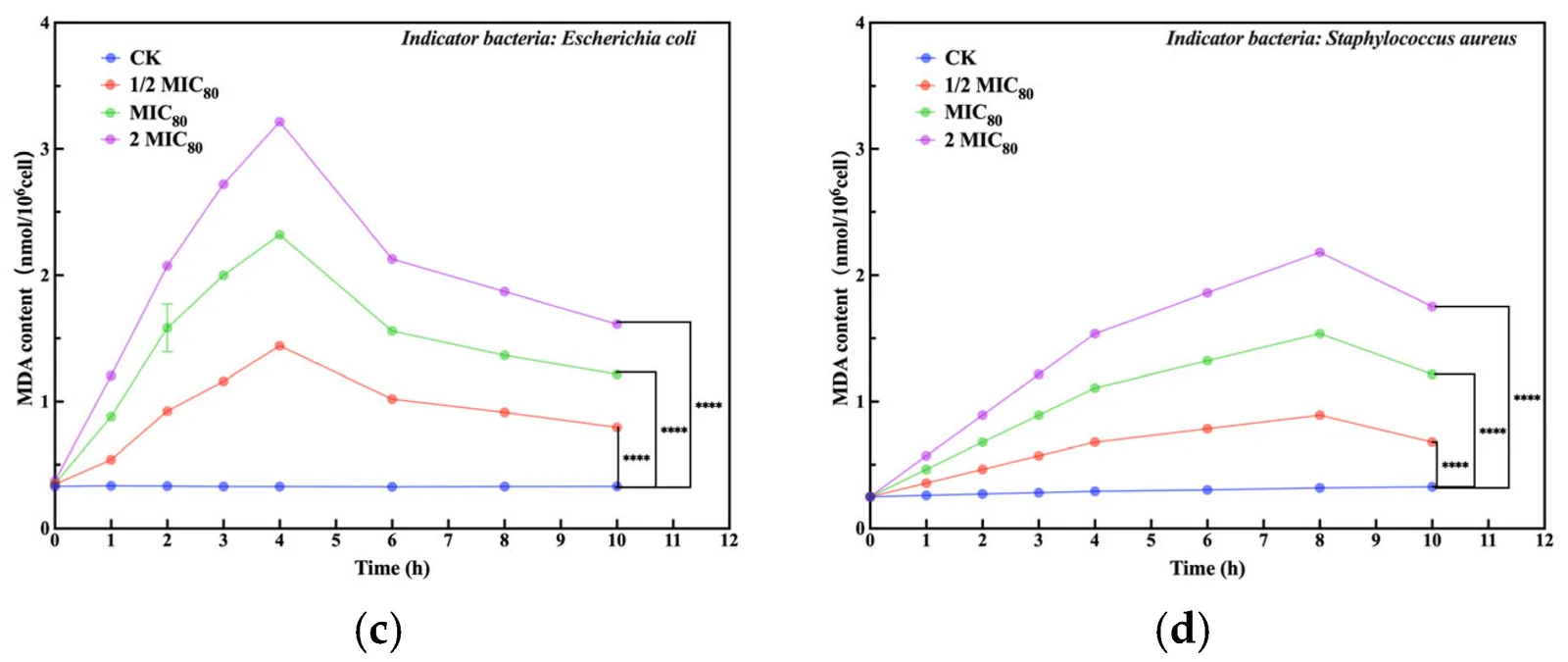
Effects of huai chrysanthemum polyphenol extract on superoxide dismutase (SOD) activity (**a**,**b**) and MDA (**c**,**d**) content in *S. aureus* and *E. coli*. (**a**,**c**) shows *E. coli*, while (**b**,**d**) shows *S. aureus*. SOD activity was expressed as U/10^4^ cells. **** indicates significant differences among the groups treated with different concentrations of chamomile polyphenols. MDA content was expressed as nmol/10^6^ cells. The same statistical analysis as described in Figure 2 was applied.

SOD activity and MDA content were determined to evaluate oxidative-stress-related responses in bacterial cells after exposure to huai chrysanthemum polyphenol extract. Superoxide dismutase (SOD) is an important antioxidant enzyme that catalyzes the conversion of superoxide anions into O_2_ and H_2_O_2_, thereby reducing oxidative damage caused by reactive oxygen species [24]. As shown in Figure 6a,b, SOD activity in the CK groups of *E. coli* and *S. aureus* remained relatively stable during incubation. In contrast, SOD activity in the treatment groups initially decreased and then increased during prolonged exposure. For *E. coli*, SOD activity increased rapidly after 4 h and peaked at 6 h, whereas *S. aureus* showed a delayed increase and reached its peak at 8 h. At the later stage of treatment, SOD activity in the 1/2MIC_80_, MIC_80_, and 2MIC_80_ groups was significantly higher than that in the CK group (*p* < 0.05), with the highest activity observed in the 2MIC_80_ group.

As shown in Figure 6c,d, MDA content in the CK groups of *E. coli* and *S. aureus* remained relatively stable during the experimental period and was consistently lower than that in the treatment groups. After treatment with huai chrysanthemum polyphenol extract, MDA content increased in a concentration-dependent manner in both bacterial strains. In *E. coli*, MDA content increased rapidly during the first 4 h and reached a peak at 4 h. At 2MIC_80_, the MDA content reached 3.21 nmol/10^6^ cells, which was 9.73-fold higher than that of the CK group. In *S. aureus*, MDA content increased more gradually and peaked at 8 h, reaching 2.18 nmol/10^6^ cells at 2MIC_80_, which was 6.81-fold higher than that of the CK group. At 10 h, MDA content in the 1/2MIC_80_, MIC_80_, and 2MIC_80_ groups was significantly higher than that in the CK group (*p* < 0.05), and the 2MIC_80_ group showed the highest level in both strains.

### 3.7. Effects of Huai Chrysanthemum Polyphenol Extract on Bacterial Morphology and Protein Profiles

**Figure 7:**
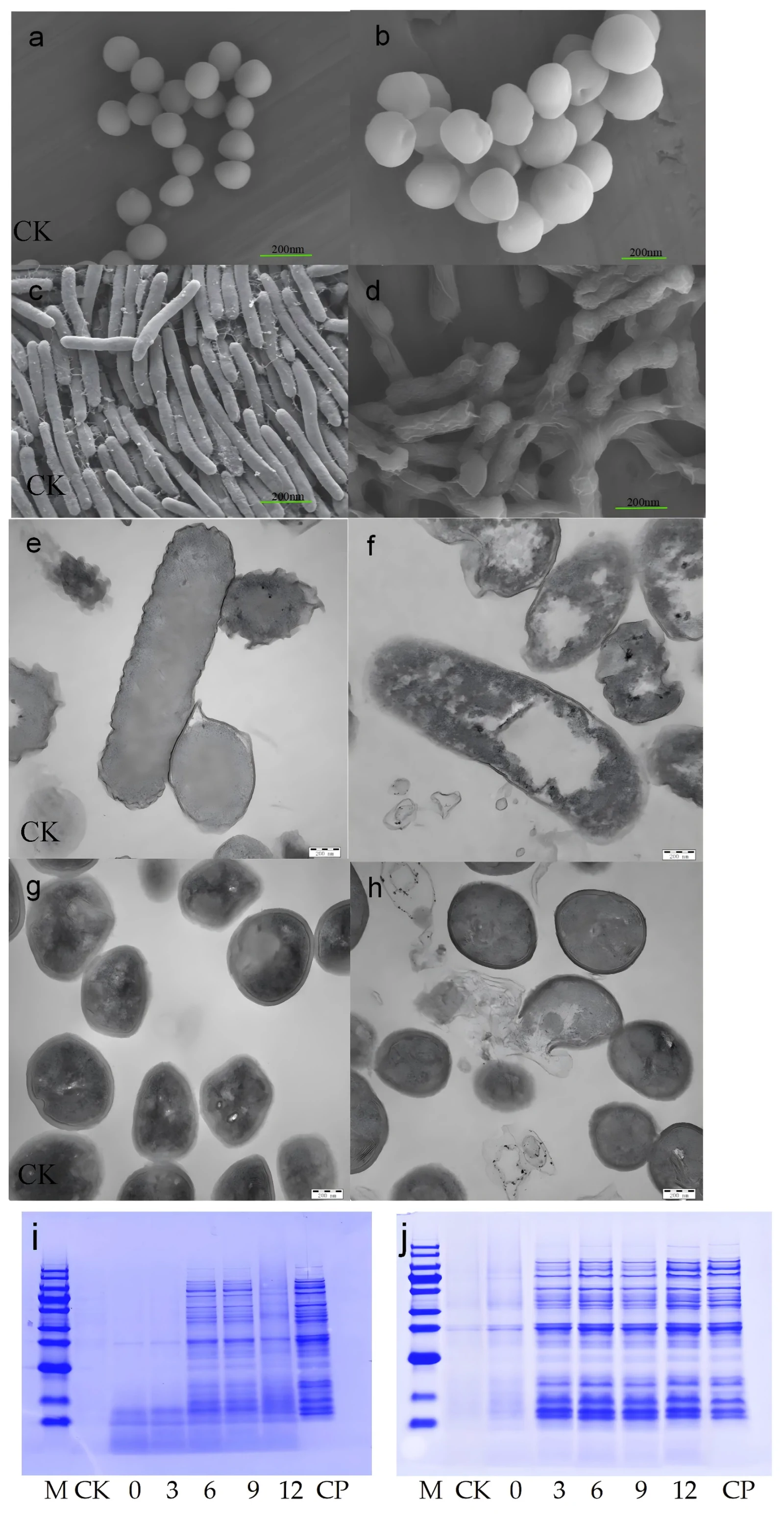
Morphological, ultrastructural, and protein-profile changes in *S. aureus* and *E. coli* after treatment with huai chrysanthemum polyphenol extract. (**a**,**b**) SEM images of untreated and extract-treated *S. aureus*; (**c**,**d**) SEM images of untreated and extract-treated *E. coli*; (**e**,**f**) TEM images of untreated and extract-treated *E. coli*; (**g**,**h**) TEM images of untreated and extract-treated *S. aureus*; (**i**,**j**) SDS-PAGE profiles of intracellular proteins from *S. aureus* and *E. coli*, respectively. M, molecular weight marker; CK, untreated control; 0, 3, 6, 9, and 12, treatment time; CP, positive control. Scale bars: 200 nm.

SEM and TEM were used to observe the morphological and ultrastructural changes in bacterial cells after treatment with huai chrysanthemum polyphenol extract. As shown in Figure 7a,c, untreated *S. aureus* and *E. coli* cells displayed regular morphology, intact cell boundaries, and relatively smooth cell surfaces. After exposure to the extract, visible morphological damage was observed in both bacterial strains, but the degree and characteristics of damage differed between them.

For *S. aureus* (Figure 7b), most cells still retained their basic spherical morphology after treatment; however, some cells showed surface roughening, slight shrinkage, local collapse, and pore-like defects. Compared with the untreated control, the treated cells exhibited less regular outlines and partially damaged surface structures. For *E. coli (*Figure 7d), the morphological changes were more pronounced. Treated cells showed obvious shrinkage, deformation, surface irregularity, membrane disruption, and pore formation. In some severely damaged cells, the cell boundary became unclear, and leakage of intracellular material was observed. These results indicated that huai chrysanthemum polyphenol extract caused visible surface damage in both Gram-positive and Gram-negative bacterial cells, with more severe morphological disruption observed in *E. coli* under the present experimental conditions.

TEM observations further confirmed the ultrastructural changes induced by the extract. Compared with untreated cells (Figure 7e,g), extract-treated bacterial cells (Figure 7f,h) showed reduced intracellular electron density, cytoplasmic dissolution, local disruption of the cell wall or membrane structure, membrane collapse, and plasmolysis-like changes. These ultrastructural alterations were consistent with the SEM observations and supported the results of extracellular protein leakage and extracellular AKP activity.

SDS-PAGE was performed to further examine changes in intracellular protein profiles after treatment with huai chrysanthemum polyphenol extract. As shown in Figure 7i,j, the untreated control groups showed clear intracellular protein bands. After treatment with the extract at the MIC_80_ level, the intensity of intracellular protein bands in both *S. aureus* and *E. coli* gradually decreased with increasing incubation time. Compared with the untreated control, extract-treated samples exhibited progressively weakened protein bands, indicating a reduction in detectable intracellular proteins. This result was consistent with the increased extracellular protein concentration observed in Figure 4, suggesting that protein loss occurred during extract treatment.

## 4. Discussion

### 4.1. Phenolic Signature and Antibacterial Relevance of Huai Chrysanthemum Polyphenol Extract

The LC–MS/MS analysis characterized huai chrysanthemum polyphenol extract as a flavonoid-rich phenolic mixture, with luteoloside as the predominant flavonoid and caffeic acid as the major phenolic acid. This profile agrees with the general phytochemical features of *Chrysanthemum morifolium*, in which flavonoids and phenolic acids are regarded as important bioactive constituents [5,6,7]. The presence of luteoloside, luteolin, apigenin, quercetin derivatives, and caffeic acid provides a chemical basis for the antibacterial activity observed in this study. Xi et al. [28] reported that luteolin inhibited *E. coli* and *S. aureus* and promoted leakage of cellular contents, which agreed with the phenolic composition and protein leakage results in the present study.

The phenolic profile of huai chrysanthemum showed clear differences from those reported for other chrysanthemum materials. Zhang et al. [29] reported marein, isookanin, and didymin as the main phenolics in Snow chrysanthemum. In “Taraneh” and “Azita” chrysanthemum cultivars, ferulic acid and chlorogenic acid were described as predominant compounds [30,31]. Prakruthi et al. [32] also identified catechin and chlorogenic acid as major phenolics in chrysanthemum samples. These comparisons indicate that huai chrysanthemum has a distinct phenolic signature, which may be shaped by cultivar, origin, and processing conditions.

Phenolic acids and flavonoids have been associated with antibacterial activity through their effects on bacterial envelope structure and cellular homeostasis [12,23]. In the present study, the extract inhibited both *E. coli* and *S. aureus*, supporting its activity against representative Gram-negative and Gram-positive bacteria. The inhibition-zone assay, MIC_80_ determination, and growth curve results formed a consistent antibacterial profile. Rather than assigning the activity to a single compound, the current results point to the combined contribution of multiple phenolic constituents in the extract.

### 4.2. Cell Envelope Perturbation as a Central Antibacterial Response

Protein leakage increased after extract treatment, showing that intracellular macromolecules were released into the culture supernatant. Similar leakage of cellular constituents has been reported in bacteria treated with plant-derived antimicrobial compounds [33,34,35]. The zeta potential shifted toward less negative values, indicating altered bacterial surface charge.

Extracellular AKP activity provided further support for cell envelope perturbation. AKP is normally retained within bacterial cells or the periplasmic region, and its release into the supernatant is commonly used as an indicator of increased envelope permeability [36]. In Gram-negative bacteria, lipopolysaccharides are essential for maintaining the outer membrane barrier [26], which may influence the interaction between phenolic compounds and bacterial cells. The concurrent changes in protein leakage, zeta potential, and extracellular AKP activity therefore suggest that the extract affected cell envelope integrity. The decrease in intracellular ATP connected the envelope-related changes with energy metabolism. Tian et al. [37] reported that protocatechualdehyde reduced intracellular ATP in Yersinia enterocolitica together with membrane damage. Xie et al. [38] found that dandelion flower phenolic extract increased extracellular ATP leakage and reduced Na^+^-K^+^ ATPase activity in *E. coli*, with caffeic acid and luteolin identified as representative phenolic compounds. In the present study, ATP reduction agreed with the leakage and AKP results, suggesting that disturbance of the bacterial envelope was accompanied by changes in energy-related cellular processes.

### 4.3. Morphological Changes and Intracellular Protein Loss After Extract Treatment

The changes in SOD activity and MDA content indicated that bacterial cells responded to huai chrysanthemum polyphenol extract with altered redox-related status. MDA accumulation is commonly associated with lipid peroxidation, while changes in SOD activity reflect the response of bacterial antioxidant defense during stress exposure. In this study, the different response patterns of *E. coli* and *S. aureus* may be related to their cell envelope structures. *E. coli* has an outer membrane and a thin peptidoglycan layer, whereas *S. aureus* has a thick peptidoglycan wall. This structural difference may partly explain the different timing and degree of SOD and MDA responses between the two bacteria. Since ROS was not directly measured, the SOD and MDA data were used to describe redox-related changes rather than ROS generation. SEM and TEM gave direct morphological evidence of cell damage after extract treatment. Untreated cells showed regular morphology and clear cell boundaries, while treated cells exhibited surface roughening, shrinkage, local collapse, membrane disruption, and leakage of intracellular material. Similar changes were reported by Wang et al. [39], who found that *Dodartia orientalis* L. essential oil caused irregular bacterial surfaces, cell rupture, and leakage of cellular contents. In the present study, the damage appeared more evident in *E. coli*, which may be associated with the presence of the outer membrane and the relatively thin peptidoglycan layer.

The SDS-PAGE results showed that intracellular protein bands became weaker after treatment with huai chrysanthemum polyphenol extract. This change agreed with the increase in extracellular protein concentration, indicating loss of intracellular proteins during treatment. Plant phenolics have been reported to affect bacterial membrane integrity and protein distribution [40]. Borges et al. [41] reported that ferulic and gallic acids showed antibacterial activity against pathogenic bacteria, and Lou et al. [42] found that chlorogenic acid caused leakage of intracellular components. These findings are consistent with the protein leakage and SDS-PAGE results in this study, suggesting that intracellular protein loss was involved in the antibacterial effect of huai chrysanthemum polyphenol extract.

## 5. Conclusions

Twenty-two polyphenolic constituents were identified in the chrysanthemum extract, among which caffeic acid and luteoloside were the most abundant. The extract exhibited clear antibacterial activity against *E. coli* and *S. aureus*, with an MIC_80_ of 12.5 mg/mL for both strains. Changes in intracellular protein content, ATP levels, zeta potential, and oxidative stress responses suggested that exposure to the extract was associated with substantial physiological disturbance in bacterial cells. Structural deformation observed by SEM further supported this conclusion. These results suggest that huai chrysanthemum polyphenol extract may inhibit bacterial growth by disturbing cellular homeostasis and compromising cell envelope integrity. These findings highlight chrysanthemum polyphenols as promising natural antimicrobial candidates for food applications. Future studies should further evaluate their safety, storage stability, and antibacterial efficacy in representative food matrices, such as meat products, beverages, or ready-to-eat foods. In addition, their effects on sensory attributes, color, flavor, and physicochemical quality should be assessed to determine suitable application forms and effective concentrations.

## Data Availability

All data included in this study are available upon request by contact with the corresponding author.
